# Genomic study of *TEX15* variants: prevalence and allelic heterogeneity in men with spermatogenic failure

**DOI:** 10.3389/fgene.2023.1134849

**Published:** 2023-05-10

**Authors:** Sidra Qureshi, Jimmaline J. Hardy, Christopher Pombar, Andrea J. Berman, Agnieszka Malcher, Tara Gingrich, Rachel Hvasta, Jannah Kuong, Sarah Munyoki, Kathleen Hwang, Kyle E. Orwig, Jawad Ahmed, Marta Olszewska, Maciej Kurpisz, Donald F. Conrad, Muhammad Jaseem Khan, Alexander N. Yatsenko

**Affiliations:** ^1^ Department of Molecular Biology and Genetics, Institute of Basic Medical Sciences, Khyber Medical University, Peshawar, Pakistan; ^2^ Department of Obstetrics, Gynecology, and Reproductive Sciences, Magee-Women’s Research Institute, University of Pittsburgh School of Medicine, Pittsburgh, PA, United States; ^3^ Department of Biological Sciences, Dietrich School of Arts and Sciences, University of Pittsburgh, Pittsburgh, PA, United States; ^4^ Institute of Human Genetics, Polish Academy of Sciences, Poznan, Poland; ^5^ Department of Urology, School of Medicine, University of Pittsburgh, Pittsburgh, PA, United States; ^6^ Department of Genetics, Oregon National Primate Research Center, Oregon Health and Science University, Beaverton, OR, United States; ^7^ Department of Pathology, School of Medicine, University of Pittsburgh, Pittsburgh, PA, United States; ^8^ Department of Genetics, School of Public Health, University of Pittsburgh, Pittsburgh, PA, United States

**Keywords:** male infertility, next-generation sequencing, non-obstructive azoospermia (NOA), oligozoospermia, TEX15

## Abstract

**Introduction:** Human spermatogenesis is a highly intricate process that requires the input of thousands of testis-specific genes. Defects in any of them at any stage of the process can have detrimental effects on sperm production and/or viability. In particular, the function of many meiotic proteins encoded by germ cell specific genes is critical for maturation of haploid spermatids and viable spermatozoa, necessary for fertilization, and is also extremely sensitive to even the slightest change in coding DNA.

**Methods:** Here, using whole exome and genome approaches, we identified and reported novel, clinically significant variants in testis-expressed gene 15 (*TEX15*), in unrelated men with spermatogenic failure (SPGF).

**Results:** TEX15 mediates double strand break repair during meiosis. Recessive loss-of-function (LOF) *TEX15* mutations are associated with SPGF in humans and knockout male mice are infertile. We expand earlier reports documenting heterogeneous allelic pathogenic *TEX15* variants that cause a range of SPGF phenotypes from oligozoospermia (low sperm) to nonobstructive azoospermia (no sperm) with meiotic arrest and report the prevalence of 0.6% of *TEX15* variants in our patient cohort. Among identified possible LOF variants, one homozygous missense substitution c.6835G>A (p.Ala2279Thr) co-segregated with cryptozoospermia in a family with SPGF. Additionally, we observed numerous cases of inferred *in trans* compound heterozygous variants in *TEX15* among unrelated individuals with varying degrees of SPGF. Variants included splice site, insertions/deletions (indels), and missense substitutions, many of which resulted in LOF effects (i.e., frameshift, premature stop, alternative splicing, or potentially altered posttranslational modification sites).

**Conclusion:** In conclusion, we performed an extensive genomic study of familial and sporadic SPGF and identified potentially damaging TEX15 variants in 7 of 1097 individuals of our combined cohorts. We hypothesize that SPGF phenotype severity is dictated by individual *TEX15* variant’s impact on structure and function. Resultant LOFs likely have deleterious effects on crossover/recombination in meiosis. Our findings support the notion of increased gene variant frequency in SPGF and its genetic and allelic heterogeneity as it relates to complex disease such as male infertility.

## Introduction

Male infertility is a complex health condition that affects 7% of reproductive aged men around the world (Krausz and Riera-Escamilla, 2019). Spermatogenic failure (SPGF) is responsible for the majority of male factor diagnoses and variably manifest in severity from reduced (oligozoospermia) to absent (azoospermia) sperm in ejaculated semen. Among the established causes of primary SPGF originating in the testes, ∼20% of patients have genetic aberrations including numerical or structural errors of chromosomal complement, microdeletions of the Y chromosome in the Azoospermia Factor (AZF) region, or commonly known mutations in autosomal genes that contribute to spermatogenesis (Reijo et al., 1996; Sarrate et al., 2005; Jungwirth et al., 2012). However, ∼40% of infertile men are negative for these established markers and other common causes like hormonal abnormalities, presenting as idiopathic (Nieschlag et al., 2011).

Recent efforts to identify pathogenic variants associated with SPGF phenotypes led to several key findings, for example, MultL Homolog 3 (*MLH3*), Kelch like family member 10 (*KLHL10*), and Zinc Finger MYND type 15 (*ZMYND15*); still the majority of cases do not have a clear diagnosis (Yatsenko et al., 2006; Ayhan, 2014; Punab et al., 2017). As such, the highly intricate nature of spermatogenesis and involvement of more than 4,000 genes warrants further genetic characterization (Jan et al., 2017). A significant proportion of SPGF diagnoses can be attributed to defects in meiosis caused by genetic mutations (Wyrwoll et al., 2020). Any disruption in the process during DNA replication, DNA double strand break (DSB) and mismatch repair, spindle formation, synapsis, recombination or chromosome segregation can lead to defects in spermatogenesis and reduction or absence of functional sperm in semen (Miyamoto et al., 2016). Recent identification of Meiosis 1 Associated Protein (*M1AP*), Testis Expressed 11 (*TEX11*), Testis Expressed 14 (*TEX14*), and Testis Expressed 15 (*TEX15*) mutations has demonstrated that biallelic and hemizygous single nucleotide variants and indels in meiotic genes play a crucial role in SPGF phenotypes (Yang et al., 2015; Yatsenko et al., 2015; Boroujeni et al., 2018; Wyrwoll et al., 2020).

For this study, we utilized whole exome and genome approaches to determine genetic causes of SPGF. Our investigation resulted in the identification of multiple *TEX15* variants in SPGF patients. *TEX15* encodes an essential meiotic protein and belongs to the family of Testis Expressed (TEX) genes expressed predominantly in germ cells (Wang et al., 2001). A high degree of sequence conservation and similarity exists between human TEX15 and other species. *TEX15* knockout mice are infertile due to meiotic arrest (Yang et al., 2008; Bellil et al., 2021). Moreover, multiple case reports have reported an association of *TEX15* variants with SPGF in humans (Okutman et al., 2015; Colombo, Pontoglio, and Bini, 2017; Wang et al., 2018; Araujo et al., 2020; Cannarella et al., 2021). Given the increasing number of cases caused by *TEX15* mutations, our aim was to identify the allelic heterogeneity and prevalence of rare, pathogenic *TEX15* variants. Here we report the prevalence and allelic heterogeneity of *TEX15* variants associated with SPGF in a combined cohort of over 1,000 infertile men.

## Material and methods

### Study cohorts

Patients were recruited from Khyber Medical University, Pakistan (n = 24), University of Pittsburgh, United States (n = 110), and Institute of Human Genetics, Polish Academy of Sciences, Poland (n = 39). We analyzed WES data from a previously unpublished cohort of 924 sporadic cases of non-obstructive azoospermia sequenced by the GEMINI Consortium (https://www.ncbi.nlm.nih.gov/pmc/articles/PMC9792524/). Informed consent was obtained from patients and participating family members after briefing about the research project. 1,033 patients within the overall SPGF cohort were diagnosed with azoospermia (no sperm in the ejaculate), 7 patients were diagnosed with cryptozoospermia (<0.1 million sperm/mL), 38 were diagnosed with severe oligozoospermia (0.1–5 million sperm/mL), and 19 patients were diagnosed with oligozoospermia (5–15 million sperm/mL) in accordance with American Urology Association and American Society for Reproductive Medicine guidelines (Schlegel et al., 2021). Patient evaluation also included serum reproductive hormone levels, karyotyping, ultrasound, and testicular biopsy (where appropriate). Individuals with a history of non-genetic causes for SPGF (e.g., trauma, surgery, or medication), obstructive azoospermia (e.g., CBAVD), abnormal sex chromosome evaluation on karyotype or Y chromosome microdeletions were excluded from the study.

### Whole exome sequencing

DNA was isolated from peripheral blood and whole exome sequencing and analysis were performed as previously described (Hardy et al., 2021). Briefly, DNA was isolated from blood with the Gentra Puregene kit (Qiagen, United States). For samples prepared in Pittsburgh (Khyber Medical University and University of Pittsburgh), whole exome sequencing libraries were constructed with the SOPHiA Whole Exome Solution (SOPHiA GENETICS, Inc., United States) and sequenced on the Illumina NovoSeq 6,000 (Novogene, Sacramento, CA). Paired-end fragments were sequenced with target read length of 150 bp and average depth coverage of ∼110x per target interval. Raw data quality obtained was evaluated with FastQC software (Babraham Bioinformatics, Babraham Institute, UK). FASTQ files were aligned with the GRCh37/hg19 reference genome using SOPHiA Genetics DDM platform (SOPHiA GENETICS, Inc., Boston, MA, United States), utilizing proprietary algorithm with BaseSpace Burroughs-Wheeler Aligner (BWA) with the BWA-MEM algorithm. Among many polymorphisms with minor allele frequencies (MAF) greater than 1%, the following variants with MAF>15% were identified at the expected frequencies across our cohort: p. Leu1724Val (gnomAD MAF: 0.18297; SPGF MAF: 0.16044), p. Asn1698Ser (gnomAD MAF: 0.18309; SPGF MAF: 0.16089), p. Ile1422Val (gnomAD MAF: 0.25998; SPGF MAF: 0.23701), p. Cys491Arg (gnomAD MAF: 0.26515; SPGF MAF: 0.24157), and p. Thr2862 = (gnomAD MAF: 0.99269; SPGF MAF: 0.99407). Since the majority of the patients were recruited without ethnic identification, we are unable to reliably ethnically match our cohort population to the gnomAD reported frequencies, this may contribute to the slight discrepancy between the observed and expected MAFs of our variants in the SPGF cohort and the general population. We have estimated matched frequency of polymorphic variants based on general population structure to validate the power of our variant detection softwares.

### Whole genome sequencing

Whole-genome sequencing (WGS) was performed on an Illumina HiSeq X (Illumina San Diego, CA) with minimum coverage of 30 × (100–120 Gb per sample) (Malcher et al., 2022). The SPGF patients from the Polish Academy of Sciences (n = 39) were sequenced with WGS methodology. Our WGS analysis did not identify new or significant deep intronic variants.

### Variant annotation

WES variants were annotated using the SOPHiA Genetics DDM (SOPHiA GENETICS, Inc., Boston, MA, United States) and Fabric Enterprise (Fabric Genomics, Oakland, CA) platforms. Variants were filtered through a list of 336 genes curated from previously reported gene candidate lists (Oud et al., 2019; Alhathal et al., 2020; Houston et al., 2021) with a moderate or above level of evidence. To infer haplotype for sporadic patients without familial segregation data, we utilized gnomAD’s phasing program, which makes use of the Expectation-Maximization algorithm to infer haplotypes of compound heterozygous variants (https://gnomad.broadinstitute.org/variant-cooccurrence) (Niu, 2004). WGS variants were annotated using Ensembl Variant Effect Predictor 30. Databases including 1,000 Genomes (NCBI browser), ESP6500 (National Heart, Lung, and Blood Institute), ExAC and gnomAD (Broad Institute) were used for identification of variant allele frequency in the general population. For evaluating potential clinical significance of variants, the following databases were used: HGMD (Stenson et al., 2014) (Qiagen) (http://www.hgmd.cf.ac.uk/ac/index.php), OMIM (Amberger et al., 2015) (Johns Hopkins University) (https://www.omim.org/), and ClinVar (NCBI) (https://www.ncbi.nlm.nih.gov/clinvar/). The MGI database (Blake et al., 2021) (Jackson Laboratory, United States) (http://www.informatics.jax.org/) was used for evaluation of gene variants from animal models. AceView (National Center for Biotechnology Information, NCBI) (https://www.ncbi.nlm.nih.gov/), GTEx Portal (Lonsdale et al., 2013) (Broad Institute) (https://gtexportal.org/home/), Human Protein Atlas (Uhlen et al., 2010) (https://www.proteinatlas.org/), and BioGPS (Wu et al., 2009) (Scripps Research Institute) (http://biogps.org/#goto=welcome) databases were used for evaluation of gene expression at the tissue level. Clustal Omega (Madeira et al., 2019) (EMBL-EBI) (https://www.ebi.ac.uk/Tools/msa/clustalo/) and PhyloP (Pollard et al., 2010) (Cornell University) (http://compgen.cshl.edu/phast/) were used for assessing conservation of variants. CADD (Kircher et al., 2014) (University of Washington, Hudson-Alpha Institute for Biotechnology and Berlin Institute of Health) (https://cadd.gs.washington.edu/), NNSplice (Berkley *Drosophila* Genome Project) (https://www.fruitfly.org/seq_tools/splice.html), PolyPhen-2 (Adzhubei et al., 2010) (Harvard University) (http://genetics.bwh.harvard.edu/pph2/), SIFT (Kumar, Henikoff, and Ng, 2009) (J. Craig Venter Institute) (https://sift.bii.a-star.edu.sg/), MaxEntScan (Yeo and Burge, 2004) (Massachusetts Institute of Technology) (http://hollywood.mit.edu/burgelab/maxent/Xmaxentscan_scoreseq.html), MutationTaster (Schwarz et al., 2010) (Charité) (https://www.mutationtaster.org/), and VVP (Flygare et al., 2018) were used for prediction of consequence of amino acid changes on protein function. The R programming environment was used to identify WES variants with causative effect for non-obstructive azoospermia within the GEMINI cohort.

### Variant confirmation

Variants obtained by WES were confirmed by Sanger sequencing using BigDye sequencing kit (ThermoFisher Scientific). *Primer3*+ software was used for designing primers. Sequencher (GeneCodes) was used for analysis of Sanger sequencing results.

### Single-cell RNA-seq data analysis

**FIGURE 1:**
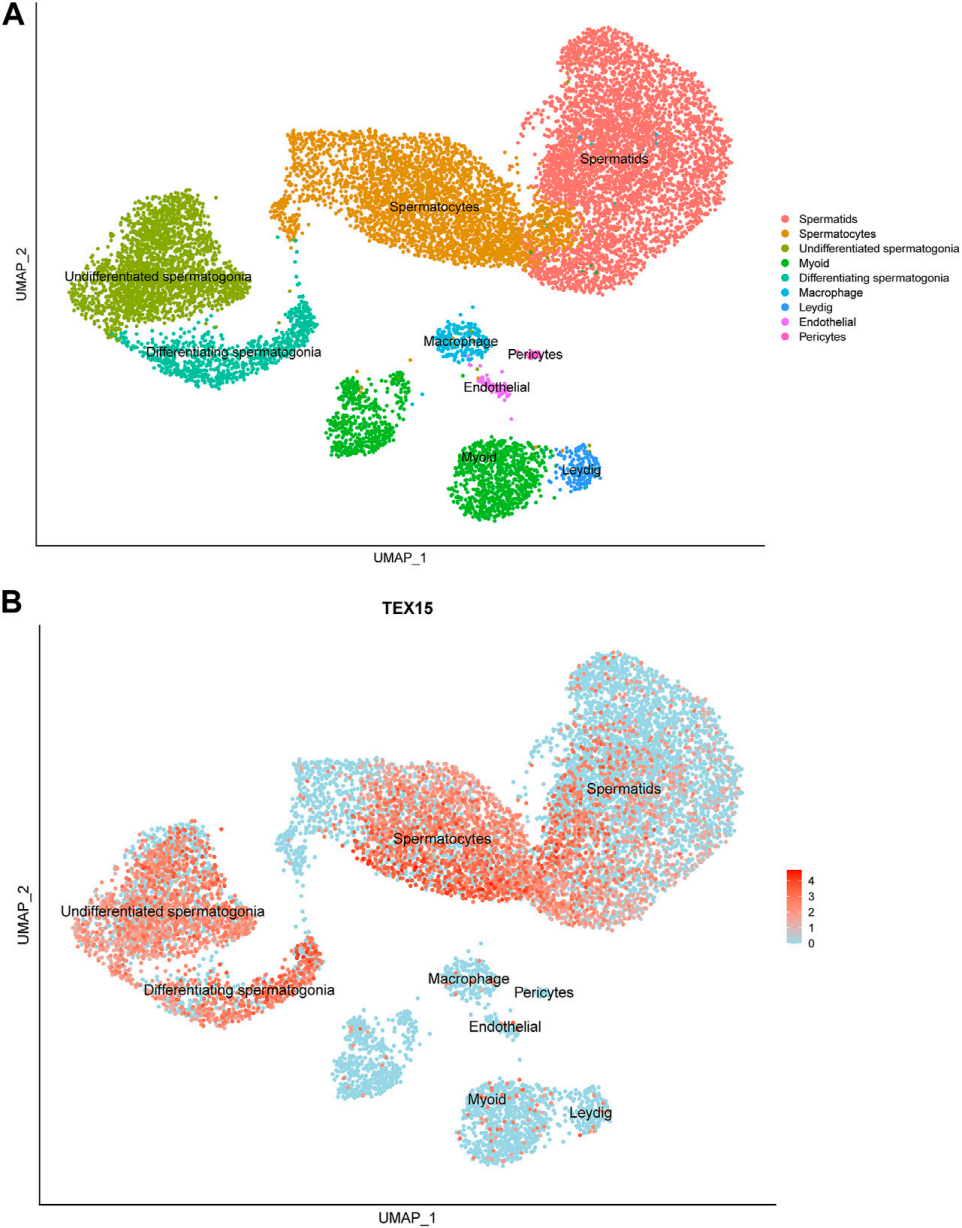
Single-cell transcriptome profiling of *TEX15* in the adult human testis. **(A)** UMAP visualization of annotated testicular cell types in the adult human testis from global clustering of 13,597 cells. Each dot represents a single testicular cell and is colored based on cell type. **(B)** UMAP plot demonstrating RNA expression pattern of *TEX15* is predominant in the human testicular germ cells. Red represents a high expression level, as shown on the color key at the bottom right.

Previously generated, processed merged human digital expression matrix data file from Drop-seq experiments on human testicular cells from 4 adult males was obtained from Gene Expression Omnibus (GEO: GSE142585) as previously described (Shami et al., 2020). The data was generated through global analysis of a total of 35,941 transcripts from 13,597 cells. The following known RNA markers for human testicular cell types were used for expression profiling: spermatogonia (GFRA1, HORMAD1, ID4, ITGA6, LY6K, STRA8, SYCP2, UCHL1, and UTF1), spermatocytes (PIWIL1 and SYCP3), spermatids (ACRV1, PRM1, TNP1, and TSSK6), Leydig cells (IFG1/2 and STAR), endothelial cells (NOSTRIN and VWF), testicular macrophages (CD52, CD163, LYZ, and TYROBP), pericytes (ADIRF, MCAM, PDGFRB, and STEAP4) or myoid cells (ACTA2 and MYH11). The normalized PCA and UMAP images developed from this data collection process were scaled and reported to support the findings in this study (Figure 1A).

### Protein modeling

The sequence of TEX15 (accession #Q9BXT5, A0A1W2PS94 isoform producing a 3,176 amino acid polypeptide) was obtained from Uniprot (UniProt Consortium, 2021). Three dimensional structural models were generated using AlphaFold (Jumper et al., 2021) and Phyre2 (Kelley et al., 2015). Because the length of TEX15 exceeds the size AlphaFold can query, we folded shorter regions for each prediction. Individual domain boundaries were initially informed by pfam (Mistry et al., 2021) and then manually curated and iteratively remodeled based on sequence alignments and structural modeling from earlier rounds of prediction. Likewise, residue sequences used to predict interdomain interactions were manually dissected and modeled with AlphaFold (Jumper et al., 2021). Structural alignment was performed in COOT (Emsley and Cowtan, 2004) and structural model figures made in PyMOL (Schrödinger, LLC).

## Results

### Homozygous TEX15 mutation cosegregates with cryptozoospermia in a Pakistani family (Case 6)

**FIGURE 2:**
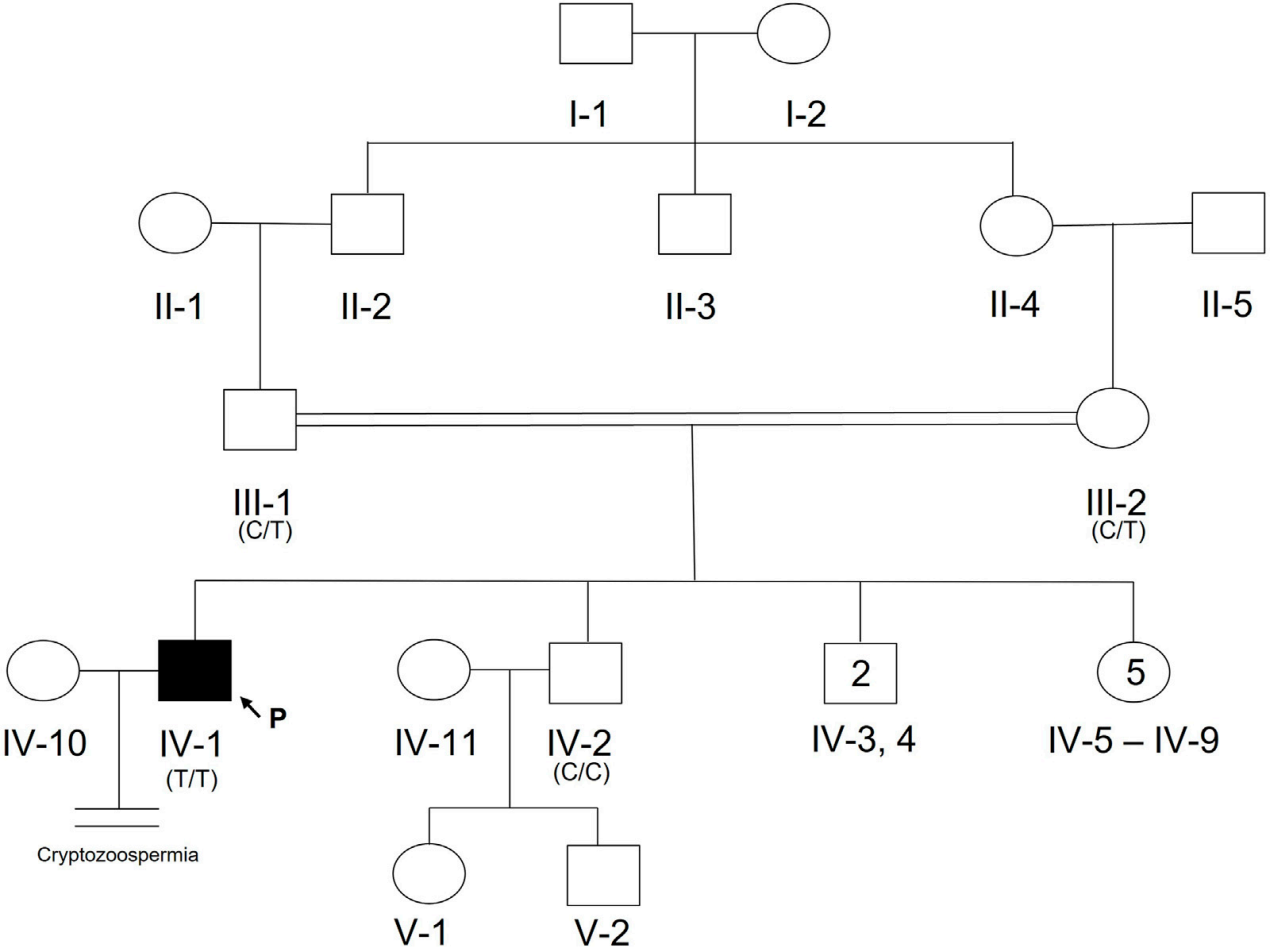
Pedigree of Pakistani family with proband presenting with cryptozoospermia and male infertility. Normal male and female members are represented with an open square or circle, respectively. The affected proband is shown with a filled symbol. A vertical line with double horizontal lines indicates an infertile couple (noted with “Cryptozoospermia” diagnosis). The arrow symbol accompanied by the letter “P” represents the proband for this family. Text in parentheses indicates TEX15 nucleotide at genomic position 8:30843344T (hg38).

A 20-year-old, married male of Pashtun ethnicity from the Khyber Pakhtunkhwa (KPK) region of Pakistan presented with unexplained infertility. The parents were first cousins and had 9 children (Figure 2). The proband’s semen analysis indicated cryptoozoospermia with normal semen volume, and viscosity (Table 1). Secondary factors affecting reproduction like ejaculatory defects, immunological irregularities, congenital disorders or environmental exposures were ruled out. Physical examination including weight, height and secondary sexual characteristics were normal. Hormonal analysis for follicle stimulating hormone, luteinizing hormone, prolactin, and testosterone all showed normal values (Table 1). T-cell cytogenetic analysis showed normal 46, XY male karyotype (Table 1). Y chromosome microdeletions as well as abnormal vas deferens associated mutations were not detected (Table 1).

**TABLE 1:** Clinical values for study participants. Ethn = ethnicity: Euro = European, N. Amer = North American, S. Asian = South Asian. Vol = semen volume; standardized to Norm (normal) according to clinical standards (Schlegel et al., 2021). Morph = normal sperm morphology. Mot = motile sperm. AZF del = AZF deletion: Neg = negative. T = Testosterone, FSH = Follicle stimulation hormone, LH = Luteinizing hormone. To account for differences in hormone testing standards, values have been normalized to the following: within range = Norm (normal), greater than clinically expected values = Elev (elevated). N/A = not available.

Case #	Ethn	Sperm conc	Vol	Morph (%)	Mot (%)	T/FSH/LH	Karyotype	AZF del
1	Euro	0/mL	Norm	0	0	Norm/Elev/Norm	46, XY	Neg
2	N. Amer	0/mL	Norm	0	0	Norm/Elev/Elev	46, XY	Neg
3	Euro	0/mL	Norm	0	0	N/A	46, XY	Neg
4	N. Amer	0/mL	Norm	0	0	Norm/Elev/Elev	46, XY	Neg
5	Euro	0/mL	Norm	0	0	N/A	46, XY	Neg
6	S. Asian	<0.1 × 10^6^/mL	Norm	50	10	Norm/Norm/Norm	46, XY	Neg
7	African	0/mL	Norm	0	0	N/A/Elev/N/A	46, XY	Neg

DNA from the proband, both parents, and one unaffected brother were subjected to WES analysis in order to identify genetic causes of SPGF. Candidates were first screened against a panel of 336 genes with evidence of association with male infertility in humans and/or mice (Oud et al., 2019; Alhathal et al., 2020; Houston et al., 2021). Among identified genes from this list, a homozygous variant in *TEX14* was ruled out due to its presence in the unaffected brother. Based on likely modes of inheritance, potential candidate variants for the family found in other relevant genes were a compound heterozygous mutation in *AKAP12*, homozygous mutation in *CHAF1B*, homozygous mutation in *KIF12*, homozygous mutation in *RIMBP3,* and hemizygous mutation in *ERCC6L.* These variants were not considered further due to overall lack of sufficient criteria (e.g., MAF>1% polymorphic in one of the ethnic subpopulations) leaving *TEX15* as the top candidate. Following thorough analysis, the homozygous *TEX15* missense variant c.6835G>A, p. Ala2279Thr identified in the proband (see **Case 6** in Table 2), was found to co-segregate with cryptozoospermia as it was not found in the unaffected brother and is heterozygous in both parents, indicating autosomal recessive inheritance (Supplementary Figure S1).

**TABLE 2:** Identified *TEX15* variants associated with SPGF. Dx = Diagnosis (NOA = Non-obstructive azoospermia, CO = cryptozoospermia). NT = nucleotide. AA = amino acid (* indicates premature stop). Zygosity = variant inheritance pattern (Het = heterozygous, Homo = homozygous). Computational predictions from SIFT (S), PolyPhen (P), PhyloP (F), MutationTaster (T), Omicia (O), CADD (C), and VVP (V) for exonic variants and NNSplice (N), MaxEntScan (M), and CADD (C) for intronic variants. B = benign, D = damaging, NA = not applicable, P = polymorphism, PD = possibly damaging, T = tolerated; ^T–^based on corresponding position in canonical transcript (e.g., c.4441G>C, p.Glu1481Gln). Minor allele frequency (MAF) values from GnomAD v3.1.2. WES = Whole Exome Sequencing; WGS = Whole Genome Sequencing. Interpretation based on ACMG technical standards and guidelines (LP = likely pathogenic; VUS = variant of uncertain significance). 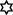
Case 2 is the only exception within the 7 identified cases with other potentially causal variants which were not able to be ruled out through our methodology as highlighted in Results and Discussion sections; further description of the potentially causal *MEI1* variants is contained in Supplementary Table S1.

Case #	Dx	NT genomic change	NT coding change	AA change	Zygosity		MAFs	Computational predictions (S, P, F, T, O C, V, or N, M, C)	WES/WGS	ACMG
1	SPGF (NOA)	g.30858836A>G	c.700–6T>C	splice region	Het		0.00041	B,B,10.02	WES	VUS
		g.30843318_30843319del	c.6860_6861del	p.Arg2287Asnfs*22	Het		0.00083	NA,NA,NA,NA,0.8,98,NA	WES	Pathogenic
2 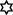	SPGF (NOA)	g.30848543T>A	c.1636A>T	p.Ile546Phe	Het		0.0001	B,B,B,B,0.099,63,10	WES	VUS
		g.30848393T>C	c.1786A>G	p.Ile596Val	Het		0.00013	B,B,B,B,0.061,19,1	WES	VUS
		g.30837478A>G	c.8820T>C	p.Ser2940Pro	Het		0	B,B,D,PD,0.199,55,13	WES	VUS
3	SPGF (NOA)	g.30847618C>A	c.2561G>T	p.Trp854Leu	Het		0	B,B,B,PD,0.183,82,6	WES	VUS
		g.30846048G>T	c.4131C>A	p.Ser1377Arg	Het		0.00001	B,D,D,PD,0.364,91,15	WES	VUS
4	SPGF (NOA)	g.30846209G>A	c.3970C>T	p.Arg1324*	Het		0.0001	D,NA,NA,B,0.621,98,36	WES	LP
		g.30845222C>T	c.4957G>A	p.Val1653Ile	Het		0.00241	B,B,B,B,0.088,11,0	WES	VUS
5	SPGF (NOA)	g.30844577C>G^T–^	c.5602G>C	p.Glu1868Gln	Het		0.00001	B,PD,D,P,NA,20.4,14	WGS	VUS
		g.30843290_30843293del	c.6886_6889del	p.Ser2296Lysfs*11	Het		0.00028	NA,NA,NA,NA,0.8,98,NA	WGS	LP
6	SPGF (CO)	g.30843344C>T	c.6835G>A	p.Ala2279Thr	Homo		0.00002	D,D,D,PD,0.482,85,18	WES	VUS
7	SPGF (NOA)	g.30842753G>A	c.7426C>T	p.Arg2476*	Het		0	D,NA,NA,D,NA,NA,NA	WES	Pathogenic
		g.30839981C>T	c.8176-17G>A	intronic	Het		0.00242	B,B,1.36	WES	VUS

**FIGURE 3:**
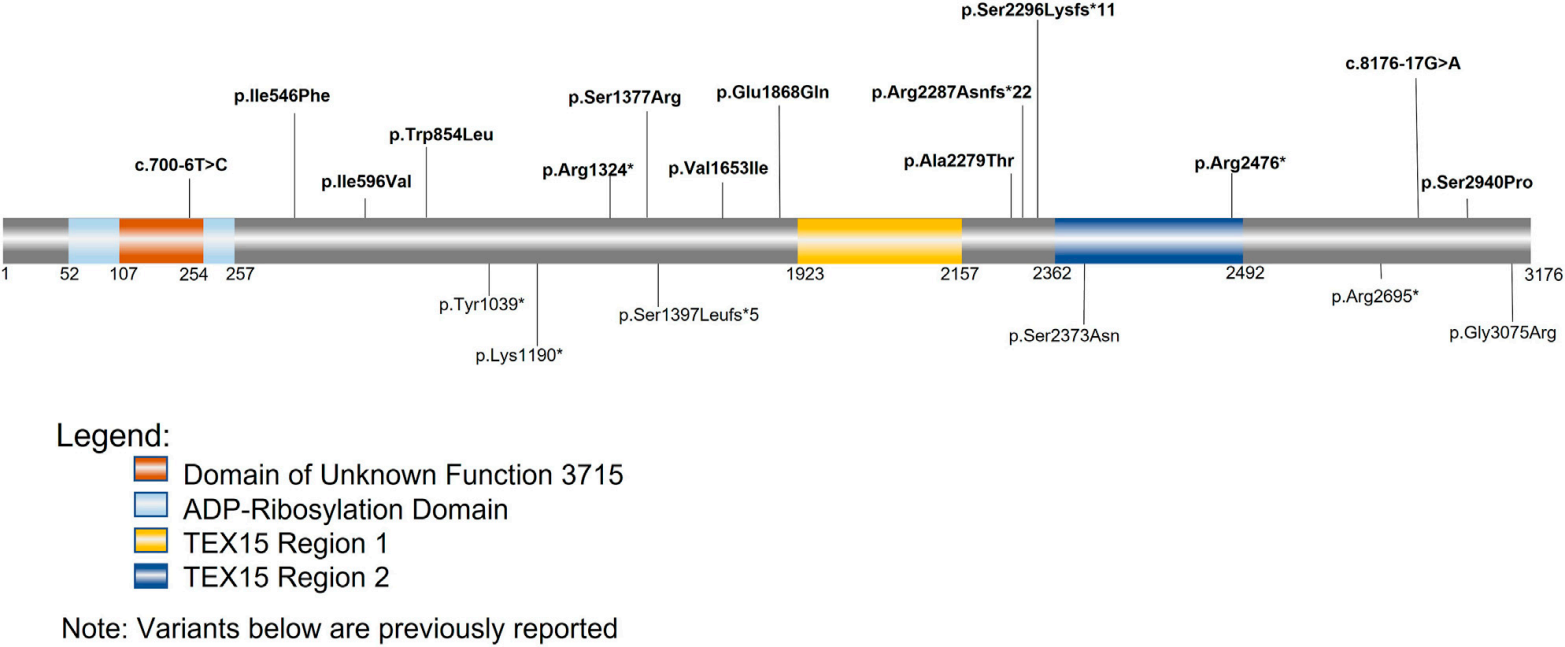
TEX15 schematic. Bolded variants listed above the sequence are variants identified within our cohorts; the variants below the sequence are variants from previously identified literature. The orange square represents the domain of unknown function 3715 (DUF3715); the overlapping light blue square represents the predicted ADP-Ribosylation domain. The two areas in yellow and dark blue represent the two TEX15 domains.

**FIGURE 4:**
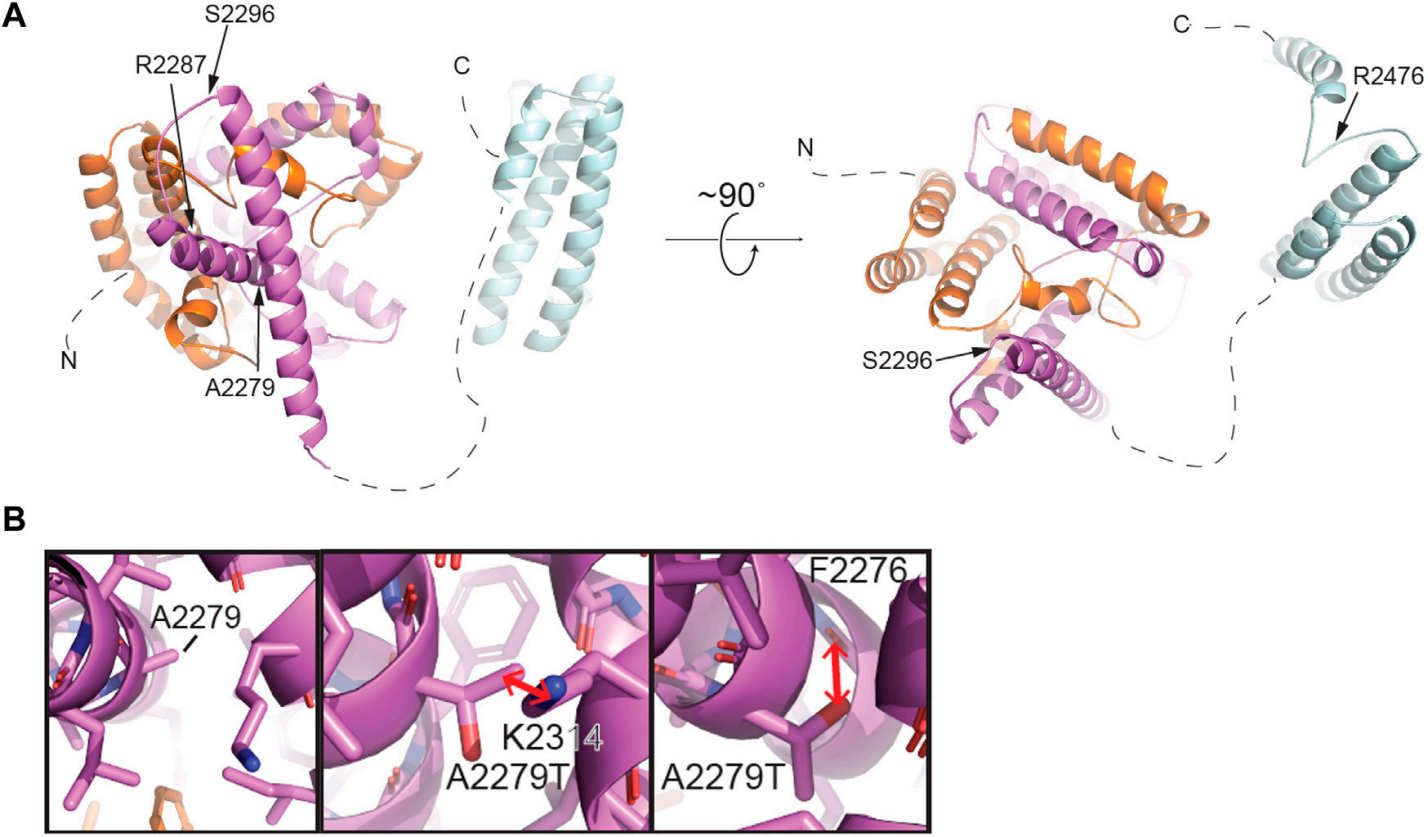
Structural modeling of TEX15 reveals some globular regions and the first partial TEX15 structural model of intrinsically disordered regions. **(A)** Structural model of the C-terminal region of TEX15 from residue 1901–2,500 generated by AlphaFold (Jumper et al., 2021). The individual TEX15 domains are colored N- to C-terminally in orange, purple, and cyan, respectively. Positions of individual mutations indicated with arrows. **(B)** Left, a zoomed view of the predicted structural environment around A2279. Right, two alternative rotamers for the A2279T mutation. Red arrows indicate steric clashes that could be introduced upon mutation of this amino acid to threonine.

Single cell RNAseq data (GEO: GSE142585) analysis from 4 adult men confirmed the expression of *TEX15* RNA in all cell types assessed, except pericytes, in adult human testes (Figure 1B) (Shami et al., 2020). *TEX15* RNA shows high expression in male germ cells including both undifferentiated and differentiating spermatogonia, spermatocytes, and spermatids. Loss of function mutations are associated with spermatogenic failure 25 (OMIM#617960 - SPERMATOGENIC FAILURE 25; SPGF25). The homozygous c.6835G>A, p. Ala2279Thr variant found in the proband affects an uncharacterized region of the 3,176 amino acid protein (ENST00000638951.1, ENSP00000492713.1) (Figure 3). Three-dimensional modeling suggests that this residue is located in a predicted alpha-helix whose position is important for interhelical packing within its TEX15 repeat (Figure 4A). Substitution of this residue with threonine results in steric clashes with surrounding amino acids; in one putative threonine rotamer, the methyl group in the side chain would clash with the side chain of lysine 2,314 (Figure 4B). In the other rotamer, the hydroxyl group of the side chain would approach the backbone of phenylalanine 2,276 too closely (Figure 4B). In each of these scenarios, the steric clash would be predicted to be propagated through the tertiary structure, destabilizing this TEX15 repeat and its interaction with the neighboring repeat. Additionally, this position is highly conserved among mice, chimpanzees, rhesus macaques, and humans (Supplementary Figure S2). These predicted steric hindrances and the relevance of position conservation are supported by the unanimously deleterious predictions in all *in silico* variant assessments performed (Table 2).

### Compound heterozygous *TEX15* coding variants are associated with varying degrees of SPGF

Follow-up analysis of WES and WGS results from the remaining 1,096 unrelated male patients (see Materials and Methods) with unexplained SPGF identified 6 cases with a total of 13 unique, potentially causative, likely *in trans* compound heterozygous/unphased deleterious *TEX15* variants (Figure 3; Table 2). A c.700–6T>C splice region variant of uncertain significance (VUS) in the N-terminal “domain of unknown function” along with a likely pathogenic c.6860_6861del, p. Arg2287Asnfs*22 frameshift resulting in a premature stop prior to the second TEX15 domain were identified in azoospermic **Case 1**; the former of which may affect a predicted ADP ribosylation site as observed with 3D modeling (Supplementary Figure S3).

**Case 2**, diagnosed with azoospermia due to late meiotic arrest, presented with 3 *TEX15* missense VUSes: c.1636A>T, p. Ile546Phe, c.1786A>G, p. Ile596Val, c.8820T>C, p. Ser2940Pro. The first variant, p. Ile546Phe, is moderately conserved (e.g., in some species instead of an isoleucine, it is a methionine or serine); none of the aligned sequences have an aromatic amino acid at this position (Supplementary Figure S2). For the second variant, p. Ile596Val, the conserved position is either isoleucine, valine, or leucine in the alignment (Supplementary Figure S2), so the small difference in the human sequence is a slight size difference, with valine being smaller. The third variant, p. Ser2940Pro, could have a strong effect on the structure of the disordered region, given the nature of proline’s side chain. The additional proline residue could lead to decreased flexibility and/or increased steric clash in the backbone. Given the similarity of the MAFs for the p. Ile546Phe and p. Ile596Val variants (.0001 and .00013 respectively), it is likely that these variants appear as *in cis* compound heterozygotes (Table 2). The novel p. Ser2940Pro variant is currently not reported in gnomAD, and as a result, we could not use gnomAD’s phasing algorithm (See Material and Methods) to predict haplotype for this variant in relation to the other two variants. However, we believe that it is possible that the p. Ile546Phe and p. Ile596Val variants are *in trans* compound heterozygotes with the PhyloP/MutationTaster software-predicted deleterious p. Ser2940Pro variant (Table 2), which may have either contributing or pathogenic associations with the azoospermic phenotype observed in the patient. This belief is supported by the notion that within Intrinsically Disordered Regions (IDRs), any position may be important for intra- or inter-molecular interactions. These computational tools are particularly suggestive as they evaluate not only amino acid substitution effect, but more specifically the effect at the relatively conserved position as mentioned above in Materials and Methods. Notably in **Case 2**, two believed *in trans* compound heterozygous (assumed on their significantly different MAF frequencies, see Materials and Methods for inference validation) Meiotic Double-Stranded Break Formation Protein 1 (*MEI1*) variants, consisting of one inframe deletion and one predicted highly deleterious missense variant, were also seen (Supplementary Table S1). *MEI1* variants (including frameshifts, non-senses, and missenses) have been previously associated with non-obstructive azoospermia in male patients (Ben Khelifa et al., 2018; Nguyen et al., 2018; Malcher et al., 2022). Given that **Case 2** is a sporadic patient without familial segregation, we are unable to confidently rule out the *MEI1* variants as potentially causal; however, without further information, they also do not have sufficient evidence for pathogenic classification than the identified *TEX15* variants, and so we retained them in **Case 2** in analysis.

**Case 3**, diagnosed with azoospermia, had a missense c.2561G>T, p. Trp854Leu substitution in addition to a c.4131C>A, p. Ser1377Arg, both of uncertain significance. The first variant, p. Trp854Leu, is a large change from a hydrophobic aromatic amino acid to a hydrophobic aliphatic amino acid. While both amino acids are classified as hydrophobic, due to the additional NH group in the tryptophan side chain, it is notably less hydrophobic than leucine. The alignment suggests that the position is not conserved for tryptophan specifically, as seen in Supplementary Figure S2, the other amino acids in that position are cystine, histidine, and asparagine, which are all polar amino acids; this is further supported by the PhyloP benign prediction (Table 2). However, again as suggested previously, due to the intrinsically disordered nature of the protein, specific position conservation is not conclusive of variant effects. The second variant, p. Ser1377Arg, is in a mostly conserved region, with only the Western clawed frog (*X. tropicalis)* and the Zebrafish (*D. rerio)* having alternative amino acids at the position: glutamine and valine respectively (Supplementary Figure S2). The change is from the tiny uncharged polar amino acid, serine, to the much larger and positively charged arginine, which could cause problems in the structure of the disordered region.

A likely pathogenic c.3970C>T, p. Arg1324* premature stop upstream of both TEX15 domains and a c.4957G>A, p. Val1653Ile VUS were found in **Case 4**, diagnosed with azoospermia as a result of early meiotic arrest. The missense variant p. Val1653Ile is in a partly conserved position; however, the position is conserved as either valine or isoleucine, which are both aliphatic amino acids, however, valine is slightly smaller (Supplementary Figure S2). From the alignment, the following amino acid is leucine for the species that have valine at the position of interest, whereas in species with isoleucine at this position, the subsequent leucine is replaced by a valine, except in *M. musculus,* where it is a histidine (Supplementary Figure S2). Given the fact that the region has unique differences in *M. musculus* compared to the others, it could mean the structure in most of the aligned species requires a small amino acid near that position.

Azoospermic **Case 5** had a c.5602G>C, p. Glu1868Gln VUS as well as a pathogenic c.6886_6889del, p. Ser2296Lysfs*11 premature termination prior to the second TEX15 domain. For the missense variant, p. Glu1868Gln, the alignment shows that the position is highly conserved for glutamic acid (Supplementary Figure S2), suggesting that the glutamic acid is important for the proper function and/or structure of the protein.

Additionally, azoospermic **Case 7** had a pathogenic c.7426C>T, p. Arg2476* premature stop in the second TEX15 domain as well as an intronic c.8276-17G>A VUS.

In addition to these compound heterozygous variants, single heterozygous variants were observed in several patients (Supplementary Table S2). Interestingly, the oligozoospermic patient **Case 18** had *in cis* compound heterozygous variants that included a pathogenic frameshift c.6860_6861del, p. Arg2287Asnfs*22 and a likely pathogenic frameshift c.6886_6889del, p. Ser2296Lysfs*11 in the uncharacterized region between the 2 TEX15 domains, both of which resulted in premature stops (Supplementary Table S2; Supplementary Figure S4). While 14 of the remaining 1,090 patients (excluding the 7 described above) observed had single or *in cis* compound heterozygous variants, these variants were classified as carrier status and overall are not thought to be pathogenic in these patients, considering *TEX15*-associated disorders are known to exhibit autosomal recessive inheritance.

## Discussion

Identifying genetic factors involved in infertility attracts growing scientific interest and requires understanding of basic molecular processes, which govern spermatogenesis and male reproduction. High-throughput genomic technologies like next-generation sequencing and comparative genomic hybridization have facilitated identification of a significant proportion of genes presumed to be essential for normal male fertility. To date, basic and translational research of male infertility has identified, with high probability, over 100 monogenic causes; those methods are based on molecular and/or clinical human phenotype, *in vivo/in vitro* modeling, and known and predicted functional associations (Houston et al., 2021). Among these genes, “strong” evidence exists for the causality of *TEX15* variants in spermatogenic failure*.* Furthermore, *TEX15* mRNA expression can be found throughout adult male testes, particularly in germ cells, while TEX15 protein is detected in spermatogonia, early spermatocytes, and post meiotic cells (Wang et al., 2001; Wang, Page, and McCarrey, 2005; Okutman et al., 2015; Boroujeni et al., 2018; Schöpp et al., 2020; Shami et al., 2020; Yang et al., 2020). Human *TEX15* is located on chromosome 8p12 and encodes for a serine rich, 3,176 amino acid protein involved in the repair of DNA DSBs during the zygonema stage of meiosis prophase I. *TEX15* expression is then temporarily downregulated in pachytene spermatocytes (Wang, Page, and McCarrey, 2005). Mouse *Tex15* expression begins in fetal male germ cells and increases postnatally (Schöpp et al., 2020; Yang et al., 2020). Male *Tex15* knockout (KO) mice are infertile due to arrest at pachynema, which inhibits the formation of haploid male gametes necessary for fertilization (Yang et al., 2008). Previously, it was proposed that TEX15 stabilizes DNA repair proteins at the time of recombination and in its absence, spermatocytes fail to develop, resulting in infertility (Yang et al., 2008). Subsequent analysis revealed that TEX15 is required for loading DSB repair proteins RAD51 and DMC1 onto recombination sites (Yang et al., 2008; Chen et al., 2016). An ensuing study further solidified the necessity of TEX15 during meiosis by establishing its role as a nuclear signal imperative for spermatocyte development (Schöpp et al., 2020; Yang et al., 2020). Additionally, *Tex15* KO mice exhibit incomplete methylation (silencing) of the promoters of transposable elements, which likely contributes to the infertility phenotype (Schöpp et al., 2020; Yang et al., 2020).

Here, using whole exome and genome approaches, we examined genomes of 1,097 men with spermatogenic failure with filters for variants from 336 previously reported gene candidates that are confidently linked to male infertility (Oud et al., 2019; Alhathal et al., 2020; Houston et al., 2021) in addition to variants in *TEX15*. After ruling out any other variants as stated in the Methods section, we identified significant *TEX15* variants (likely pathogenic and VUS) in 7 out of 1,097 men with unexplained male infertility. Given recent studies (Aston, 2014; Zhang et al., 2015; Tüttelmann et al., 2018; Cerván-Martín et al., 2020; Cioppi, Rosta, and Krausz, 2021; Ghadirkhomi et al., 2022; Guzmán-Jiménez et al., 2022), we also sought to determine if *TEX15* polymorphisms could be used as markers for infertility; however, given that the identified polymorphisms (see Materials and Methods) appeared at the expected frequency among both our control and affected populations, we found no apparent, statistically significant relationship between *TEX15* polymorphisms and SPGF.

In **Case 2**, which featured likely causal *TEX15* and *MEI1* variants, we cannot rule out the possibility of digenic effect resulting in the severe azoospermic phenotype seen in the patient, especially when considering the variable phenotypes that *TEX15* variants have been associated with. Notable *TEX15* variants include a possible splice site acceptor change c.700–6T>C in the TEX15 DUF3715 region in conjunction with a c.6860_6861del, p. Arg2287Asnfs*22 frameshift that likely eliminates the second TEX15 domain in **Case 1;** both of which potentially lead to loss of function as a result of protein truncation. The DUF3715 domain is also known to be involved in regulation of transposable elements silencing (Douse et al., 2020). We believe that TEX15 may interact with MIWI2 and MILI (Schöpp et al., 2020; Yang et al., 2020). MIWI2 is a protein required for piRNA-directed DNA methylation of transposons that is essential for derivation of germ line cells from somatic cells during fetal development, (Schöpp et al., 2020). DUF3715 also has strong similarities to a poly ADP-ribose polymerase (PARP) catalytic domain and may help facilitate RNA binding (Douse et al., 2020). Acknowledging these known associations suggests any disruption within this region could lead to loss of function and impaired spermatogenesis. Within other known domains, a premature stop was observed in the second TEX15 region in **Case 7**. Frameshifts and/or premature stops were also found in uncharacterized IDR-like regions in both oligozoospermic and azoospermic individuals highlighting the allelic heterogeneity of SPGF. Additionally, multiple patients had variants affecting amino acids with known function in post-translational modification. For instance, serine, a residue that is often acetylated, glycosylated, or phosphorylated, was replaced in several patients. Additionally, arginine, which when methylated regulates many cellular processes, was affected in multiple cases. The remaining identified variants likely alter the native secondary structure and subsequent tertiary conformation, ultimately leading to protein destabilization. TEX15 is predicted to be largely disordered, complicating the interpretation of the structural consequences of several mutations of interest. However, we successfully generated the structures of predicted globular domains based on their sequence conservation. The C-terminus of TEX15 is predicted by pfam to contain two TEX15 domains (Letunic, Khedkar, and Bork, 2021; Mistry et al., 2021; UniProt Consortium, 2021). However, AlphaFold predicted three alpha-helical bundles (Jumper et al., 2021). The first two intercalate with each other to form a ‘superdomain,’ including residues 1901–2,160; the second predicted TEX15 domain is comprised of an independent helical bundle, which could theoretically interact with the existing ‘superdomain’ (Figure 4 and Supplementary Figure S5). Based on its unrefined structural resemblance to the other two modeled TEX15 domains, it is possible that this C-terminal TEX15 domain could interact with other less characterized TEX15 domains. Indeed, sequence alignments suggest that the three observed TEX15 domains could contain conserved sequence motifs (Supplementary Figure S5). Thus, the predicted three-dimensional structure of the C-terminal region of TEX15 provides rationale for the mechanistic studies of the gene variants. Specifically, multiple observed variants result in truncation of portions of these globular domains, which could affect inter- and intra-molecular interactions. The identified conservative putative motifs in the TEX15 domain (Supplementary Figure S5B) are likely critical for structural stabilization and their disruption could interfere with the conformational fold of TEX15. We recognize that the assessment of variant effect within these putative domains and intrinsically disordered regions is constrained by the current lack of existing data on the TEX15 functional pathway; further investigation into this pivotal pathway through functional characterization assays will be necessary to discern individual variant effects on protein-protein interactions.

In conclusion, we identified unique and rare significant *TEX15* variants that likely contribute to spermatogenic failure in 0.6% of our study cohort. Considering a model with an equal gene contribution of the 1,378 known testis-specific proteins to the SPGF load, one would expect a prevalence of 0.07% (Fagerberg et al., 2014). Here, we observed a nearly 10-fold increase, which is greater than that of other germ cell specific genes, i.e., *GCNA* (<0.4%) but less than that predicted for *TEX11* (∼2%) (Yang et al., 2015; Yatsenko et al., 2015; Hardy et al., 2021). These results highlight the genetic heterogeneity of SPGF. To our knowledge, there is limited information about monogenic prevalence in male infertility, therefore, continued emphasis on improving the overall knowledge of the prevalence of other key monogenic causes would greatly advance clinical comprehensive genetic testing and aid in conclusive clinical diagnosis and treatment of male infertility.

## Figures and Tables

**Table:** 

**ACMG**	American college of medical genetics and genomics
**AZF**	Azoospermia factor
**PARP**	ADP-ribose polymerase
**CBAVD**	congenital bilateral absence of vas deferens
**CO**	Cryptozoospermia
**DNA**	deoxyribonucleic acid
**DSB**	double strand break
**GEMINI**	Genetics of male infertility initiative
**GATK**	Genome analysis toolkit
**HGMD**	Human gene mutation database
**HUSH**	Human silencing hub
**IDR**	Intrinsically disordered region
**KO**	knockout
**KPK**	Khyber pakhtunkhwa
**LOF**	loss of function
**MAF**	minor allele frequency
**NCBI**	National center for biotechnology information
**NOA**	Non-obstructive azoospermia
**OS**	Oligozoospermia
**OMIM**	Online mendelian inheritance in man
**PIWI**	P-Element induced wimpy testis
**PolyPhen**	Polymorphism phenotyping
**RNA**	ribonucleic acid
**SOS**	Severe oligozoospermia
**SPGF**	spermatogenic failure
**SIFT**	Sorting intolerant from tolerant
**TASOR**	Transcription activation suppressor
**TEX**	testis expressed
**VUS**	variant of uncertain significance
**WES**	Whole exome sequencing
**WGS**	Whole genome sequencing

## Data Availability

The data presented in the study are deposited into the ClinVar repository, accession number SUB12865746.

## Supplementary material

SUPPLEMENTARY FIGURE S1 Inheritance pattern of TEX15 g.30843344C>T (found in Case 6). **(A)** Heterozygous father. **(B)** Heterozygous mother. **(C)** Homozygous proband. **(D)** Not present in unaffected brother. Image produced with Integrative Genomics Viewer (IGV).

SUPPLEMENTARY FIGURE S2 Conservation alignment of TEX15 transcripts across 10 species. From top to bottom: Homo sapiens (H. Sapiens) = human, Pan troglodytes (P. troglodytes) = chimpanzee, Gorilla gorilla (G. gorilla) = gorilla, Macaca mulatta (M. mulatta) = rhesus macaque, Canis lupus familiaris (C. lupus familiaris) = dog, Felis Catus (F. catus) = cat, Acinonyx jubatus (A. jubatus) = cheetah, Mus musculus (M. musculus) = mouse, Xenopus tropicalis (X. tropicalis) = western clawed frog, Danio rerio (D. rerio) = zebrafish. As specified in the key, orange box = Case 1, light blue box = Case 2, purple box = Case 3, dark blue box = Case 4, grey box = Case 5, pink box = Case 6, and yellow box = Case 7. Multiple sequence alignment produced using Clustal O v1.2.4 (Sievers et al. 2011).

SUPPLEMENTARY FIGURE S3 Structural modelling of the N-terminal domain of TEX15. Structural prediction of the DUF375 generated by Phyre2.

SUPPLEMENTARY FIGURE S4 In cis compound heterozygous TEX15 variant detected in azoospermic patient Case 18. Image produced with Integrative Genomics Viewer (IGV). Shown here, the variants do not appear on alternative reads, indicating they are likely on the same chromosome, and therefore are likely single heterozygous.

SUPPLEMENTARY FIGURE S5 Examination of the structural predictions for the C-terminal TEX15 repeats. **(A)** Model interpretation statistics generated for the modeling in Figure 3 of the TEX15 domain-containing C-terminus. Left, the predicted LDDT is a measure of confidence in the model. In this experiment, the query sequence contained TEX15 residues 1901-2500, indicated on the x-axis as residues 0-599. Right, the confidence in inter-domain predictions. The darker the green, the higher the confidence in the relative positions of the residues. The predicated aligned error shows that the position and orientation of the modeling of the first two TEX15 repeats relative to the third is not accurate. **(B)** Sequence alignment generated by CLUSTAL Omega (Sievers et al. 2011) indicates there might be two conserved sequence motifs in the TEX15 repeat. **(C,D)** Mapping of the position of putative motifs 1 **(C)** and 2 **(D)** on the structure calculated by AlphaFold (Jumper et al. 2021).

SUPPLEMENTARY TABLE S1 MEI1 variants in Case 2. Dx = Diagnosis (NOA = non-obstructive azoospermia). NT = nucleotide. AA = amino acid. Computational predictions from SIFT (S), PolyPhen (P), PhyloP (F), MutationTaster (T), Omicia (O), CADD (C), and VVP (V) for exonic. D = damaging and N/A=not applicable. Minor allele frequency (MAF) values from GnomAD v3.1.2. Interpretation based on ACMG technical standards and guidelines (LB=likely benign and LP=likely pathogenic).

SUPPLEMENTARY TABLE S2 Monoallelic TEX15 variants observed in men with SPGF. Dx = Diagnosis (NOA = non-obstructive azoospermia; OS = oligozoospermia; SOS = Severe Oligozoospermia). NT = nucleotide. AA = amino acid. Computational predictions from SIFT (S), PolyPhen (P), PhyloP (F), MutationTaster (T), Omicia (O), CADD (C), and VVP (V) for exonic variants (B = benign, D = damaging, N/A = not applicable, NF = not found, NI = not identified, P = polymorphism, PD = possibly damaging, T = tolerated); eg., based on corresponding position in canonical transcript c.4441G>C, p.Glu1481Gln). Minor allele frequency (MAF) values from GnomAD v3.1.2. WES = Whole Exome Sequencing; WGS = Whole Genome Sequencing. Interpretation based on ACMG technical standards and guidelines (LB = likely benign, LP = likely pathogenic, VUS = variant of uncertain significance). *premature stop; **in cis compound heterozygous.

## References

[B1] Adzhubei I. A. Schmidt S. Peshkin L. Ramensky V. E. Anna G. Peer B. (2010). 'A method and server for predicting damaging missense mutations. Nat. methods 7, 248–249. 10.1038/nmeth0410-248 20354512 PMC2855889

[B2] Alhathal N. Maddirevula S. Coskun S. Alali H. Assoum M. Morris T. (2020). 'A genomics approach to male infertility. Genet. Med. 22, 1967–1975. 10.1038/s41436-020-0916-0 32719396

[B3] Amberger J. S. Bocchini C. A. Schiettecatte F. Scott A. F. Ada H. (2015). 'OMIM. org: Online Mendelian Inheritance in Man (OMIM®), an online catalog of human genes and genetic disorders. Nucleic acids Res. 43, D789–D798. 10.1093/nar/gku1205 25428349 PMC4383985

[B4] Araujo T. F. Friedrich C. Grangeiro C. H. P. Martelli L. R. Grzesiuk J. D. Emich J. (2020). 'Sequence analysis of 37 candidate genes for male infertility: Challenges in variant assessment and validating genes. Andrology 8, 434–441. 10.1111/andr.12704 31479588

[B5] Aston K. I. (2014). Genetic susceptibility to male infertility: News from genome-wide association studies. Andrology 2, 315–321. 10.1111/j.2047-2927.2014.00188.x 24574159

[B6] Ayhan Ö. Balkan M. Guven A. Hazan R. Atar M. Tok A. (2014). Truncating mutations in TAF4B and ZMYND15 causing recessive azoospermia. Truncating Mutat. TAF4B ZMYND15 causing recessive azoospermia 51, 239–244. 10.1136/jmedgenet-2013-102102 24431330

[B7] Bellil H. Ghieh F. Hermel E. Mandon-Pepin B. François J. (2021). Human testis-expressed (TEX) genes: A review focused on spermatogenesis and male fertility. Basic Clin. Androl. 31, 1–15. 10.1186/s12610-021-00127-7 33882832 PMC8061069

[B8] Ben Khelifa M. Ghieh F. Boudjenah R. Hue C. Fauvert D. Dard R. (2018). 'A MEI1 homozygous missense mutation associated with meiotic arrest in a consanguineous family. Hum. Reprod. 33, 1034–1037. 10.1093/humrep/dey073 29659827

[B9] Blake J. A. Baldarelli R. Kadin J. A. Richardson J. E. Smith C. L. Bult C. J. (2021). 'Mouse genome database (MGD): Knowledgebase for mouse-human comparative biology. Nucleic Acids Res. 49, D981–D987. 10.1093/nar/gkaa1083 33231642 PMC7779030

[B10] Boroujeni P. B. Sabbaghian M. Totonchi M. Sodeifi N. Sarkardeh H. Samadian A. (2018). 'Expression analysis of genes encoding TEX11, TEX12, TEX14 and TEX15 in testis tissues of men with non-obstructive azoospermia. JBRA Assist. Reprod. 22, 185–192. 10.5935/1518-0557.20180030 29932616 PMC6106636

[B11] Cannarella R. Condorelli R. A. Paolacci S. Barbagallo F. Guerri G. Bertelli M. (2021). 'Next-generation sequencing: Toward an increase in the diagnostic yield in patients with apparently idiopathic spermatogenic failure. Asian J. Androl. 23, 24–29. 10.4103/aja.aja_25_20 32655042 PMC7831827

[B12] Cerván-Martín M. Castilla J. A. Palomino-Morales R. J. Carmona F. D. (2020). 'Genetic landscape of nonobstructive azoospermia and new perspectives for the clinic. J. Clin. Med. 9, 300. 10.3390/jcm9020300 31973052 PMC7074441

[B13] Chen S. R. Hao X. X. Zhang Y. Deng S. L. Wang Z. P. Wang Y. Q. (2016). 'Androgen receptor in Sertoli cells regulates DNA double-strand break repair and chromosomal synapsis of spermatocytes partially through intercellular EGF-EGFR signaling. Oncotarget 7, 18722–18735. 10.18632/oncotarget.7916 26959739 PMC4951324

[B15] Cioppi F. Rosta V. Krausz C. (2021). 'Genetics of azoospermia. Int. J. Mol. Sci. 22, 3264. 10.3390/ijms22063264 33806855 PMC8004677

[B16] Colombo R. Pontoglio A. Bini M. (2017). 'Two novel TEX15 mutations in a family with nonobstructive azoospermia. Gynecol. Obstet. Invest. 82, 283–286. 10.1159/000468934 28355598

[B17] Douse C. H. Tchasovnikarova I. A. Timms R. T. Protasio A. V. Seczynska M. Prigozhin D. M. (2020). 'TASOR is a pseudo-PARP that directs HUSH complex assembly and epigenetic transposon control. Nat. Commun. 11, 4940. 10.1038/s41467-020-18761-6 33009411 PMC7532188

[B18] Emsley P. Cowtan K. (2004). 'Coot: Model-building tools for molecular graphics. Acta Crystallogr. D. Biol. Crystallogr. 60, 2126–2132. 10.1107/S0907444904019158 15572765

[B19] Fagerberg L. Hallström B. M. Oksvold P. Kampf C. Djureinovic D. Odeberg J. (2014). 'Analysis of the human tissue-specific expression by genome-wide integration of transcriptomics and antibody-based proteomics. Mol. Cell Proteomics 13, 397–406. 10.1074/mcp.M113.035600 24309898 PMC3916642

[B20] Flygare S. Hernandez E. J. Phan L. Moore B. Li M. Fejes A. (2018). 'The VAAST variant prioritizer (VVP): Ultrafast, easy to use whole genome variant prioritization tool. BMC Bioinforma. 19, 57. 10.1186/s12859-018-2056-y PMC5819680 29463208

[B21] Ghadirkhomi E. Angaji S. A. Khosravi M. Mashayekh M. R. (2022). Correlation of novel single nucleotide polymorphisms ofUSP26, TEX15, and TNP2 genes with male infertility in north west of Iran. Int. J. Fertil. Steril. 16, 10–16. 10.22074/IJFS.2021.521138.1058 35103426 PMC8808250

[B22] Guzmán-Jiménez A. González-Muñoz S. Cerván-Martín M. Rivera-Egea R. Garrido N. Luján S. (2022). 'Contribution of TEX15 genetic variants to the risk of developing severe non-obstructive oligozoospermia. Front. Cell Dev. Biol. 10, 1089782. 10.3389/fcell.2022.1089782 36589743 PMC9797780

[B24] Hardy J. J. Wyrwoll M. J. McFadden W. Malcher A. Rotte N. Pollock N. C. (2021). 'Variants in GCNA, X-linked germ-cell genome integrity gene, identified in men with primary spermatogenic failure. Hum. Genet. 140, 1169–1182. 10.1007/s00439-021-02287-y 33963445 PMC8266742

[B25] Houston B. J. Riera-Escamilla A. Wyrwoll M. J. Salas-Huetos A. Xavier M. J. Nagirnaja L. (2021). A systematic review of the validated monogenic causes of human male infertility: 2020 update and a discussion of emerging gene-disease relationships. Hum. Reprod. Update 28, 15–29. 10.1093/humupd/dmab030 34498060 PMC8730311

[B26] Jan S. Z. Vormer T. L. Jongejan A. Röling M. D. Silber S. J. de Rooij D. G. (2017). Unraveling transcriptome dynamics in human spermatogenesis. Development 144, 3659–3673. 10.1242/dev.152413 28935708 PMC5675447

[B27] Jumper J. Evans R. Pritzel A. Green T. Figurnov M. Ronneberger O. (2021). 'Highly accurate protein structure prediction with AlphaFold. Nature 596, 583–589. 10.1038/s41586-021-03819-2 34265844 PMC8371605

[B28] Jungwirth A. Giwercman A. Herman T. Diemer T. Kopa Z. Dohle G. (2012). European association of Urology guidelines on male infertility: The 2012 update. Eur. Assoc. Urology Guidel. Male Infertil. 2012 update 62, 324–332. 10.1016/j.eururo.2012.04.048 22591628

[B29] Kelley L. A. Mezulis S. Yates C. M. Wass M. N. Sternberg M. J. (2015). The Phyre2 web portal for protein modeling, prediction and analysis. Nat. Protoc. 10, 845–858. 10.1038/nprot.2015.053 25950237 PMC5298202

[B30] Kircher M. Witten D. M. Jain P. O'Roak B. J. Cooper G. M. Shendure J. (2014). 'A general framework for estimating the relative pathogenicity of human genetic variants. Nat. Genet. 46, 310–315. 10.1038/ng.2892 24487276 PMC3992975

[B31] Krausz C. Riera-Escamilla A. (2019). Monogenic forms of male infertility. Exp. Suppl. 111, 341–366. 10.1007/978-3-030-25905-1_16 31588539

[B32] Kumar P. Henikoff S. Ng P. C. (2009). Predicting the effects of coding non-synonymous variants on protein function using the SIFT algorithm. Nat. Protoc. 4, 1073–1081. 10.1038/nprot.2009.86 19561590

[B33] Letunic I. Khedkar S. Bork P. (2021). 'SMART: Recent updates, new developments and status in 2020. Nucleic Acids Res. 49, D458–D460. 10.1093/nar/gkaa937 33104802 PMC7778883

[B34] Lonsdale J. Thomas J. Salvatore M. Phillips R. Lo E. Shad S. (2013). The genotype-tissue expression (GTEx) project. Nat. Genet. 45, 580–585. 10.1038/ng.2653 23715323 PMC4010069

[B35] Madeira F. Young M. P. Lee J. Nicola B. Gur T. Madhusoodanan N. (2019). The EMBL-EBI search and sequence analysis tools APIs in 2019. Nucleic acids Res. 47, W636–W41. 10.1093/nar/gkz268 30976793 PMC6602479

[B36] Malcher A. Stokowy T. Berman A. Olszewska M. Jedrzejczak P. Sielski D. (2022). 'Whole-genome sequencing identifies new candidate genes for nonobstructive azoospermia. Andrology 10, 1605–1624. 10.1111/andr.13269 36017582 PMC9826517

[B37] Mistry J. Chuguransky S. Williams L. Qureshi M. Salazar G. A. Sonnhammer E. L. L. (2021). 'Pfam: The protein families database in 2021. Nucleic Acids Res. 49, D412–D419. 10.1093/nar/gkaa913 33125078 PMC7779014

[B38] Miyamoto T. Bando Y. Koh E. Tsujimura A. Miyagawa Y. Iijima M. (2016). A PLK4 mutation causing azoospermia in a man with Sertoli cell-only syndrome. Andrology 4, 75–81. 10.1111/andr.12113 26452337

[B39] Nguyen N. M. P. Ge Z. J. Reddy R. Fahiminiya S. Sauthier P. Bagga R. (2018). 'Causative mutations and mechanism of androgenetic hydatidiform moles. Am. J. Hum. Genet. 103, 740–751. 10.1016/j.ajhg.2018.10.007 30388401 PMC6218808

[B40] Nieschlag E. H Behre S. N. Ahlen H. J. (2011). 'Male reproductive health and dysfunction', 51: 751.

[B41] Niu T. (2004). 'Algorithms for inferring haplotypes. Genet. Epidemiol. 27, 334–347. 10.1002/gepi.20024 15368348

[B42] Okutman O. Muller J. Baert Y. Serdarogullari M. Gultomruk M. Piton A. (2015). Exome sequencing reveals a nonsense mutation in TEX15 causing spermatogenic failure in a Turkish family. Hum. Mol. Genet. 24, 5581–5588. 10.1093/hmg/ddv290 26199321

[B43] Oud M. S. Volozonoka L. Smits R. M. Vissers L. Ramos L. Veltman J. A. (2019). 'A systematic review and standardized clinical validity assessment of male infertility genes. Hum. Reprod. 34, 932–941. 10.1093/humrep/dez022 30865283 PMC6505449

[B44] Pollard K. S. Hubisz M. J. KateRosenbloom R. Adam S. (2010). Detection of nonneutral substitution rates on mammalian phylogenies. Genome Res. 20, 110–121. 10.1101/gr.097857.109 19858363 PMC2798823

[B45] Punab M. Poolamets O. Paju P. Vihljajev V. Pomm K. Ladva R. Korrovits P. Human reproduction laan. Hum. Reprod., 2017. 'causes of male infertility: A 9-year prospective monocentre study on 1737 patients with reduced total sperm counts', 32: 18–31. 10.1093/humrep/dew284 PMC5165077 27864361

[B46] Reijo R. RaajiAlagappan K. Page D. C. The Lancet Patrizio P. J. (1996). 'Severe oligozoospermia resulting from deletions of azoospermia factor gene on Y chromosome. Lancet 347, 1290–1293. 10.1016/s0140-6736(96)90938-1 8622504

[B47] Sarrate Z. Blanco J. Anton E. Egozcue S. Vidal F. (2005). FISH studies of chromosome abnormalities in germ cells and its relevance in reproductive counseling. J Asian J. Androl. Vidal. 7: 227–236. 10.1111/j.1745-7262.2005.00061.x 16110350

[B48] Schlegel P. N. Sigman M. Collura B. De Jonge C. J. Eisenberg M. L. Lamb D. J. (2021). Diagnosis and treatment of infertility in men: AUA/ASRM guideline part I. Fertil. Steril. 115, 54–61. 10.1016/j.fertnstert.2020.11.015 33309062

[B49] Schöpp T. Zoch A. Berrens R. V. Auchynnikava T. Kabayama Y. Vasiliauskaitė L. (2020). 'TEX15 is an essential executor of MIWI2-directed transposon DNA methylation and silencing. Nat. Commun. 11, 3739. 10.1038/s41467-020-17372-5 32719317 PMC7385494

[B50] Schwarz J. M. Rödelsperger C. Schuelke M. Seelow D. (2010). 'MutationTaster evaluates disease-causing potential of sequence alterations. Nat. methods 7, 575–576. 10.1038/nmeth0810-575 20676075

[B51] Shami A. N. Zheng X. Munyoki S. K. Ma Q. Manske G. L. Green C. D. (2020). 'Single-Cell RNA sequencing of human, macaque, and mouse testes uncovers conserved and divergent features of mammalian spermatogenesis. Dev. Cell 54, 529–547. 10.1016/j.devcel.2020.05.010 32504559 PMC7879256

[B52] Sievers F. Wilm A. Dineen D. Gibson T. J. Karplus K. Li W. (2011). 'Fast, scalable generation of high-quality protein multiple sequence alignments using Clustal Omega. Mol. Syst. Biol. 7, 539. 10.1038/msb.2011.75 21988835 PMC3261699

[B53] Stenson P. D. Mort M. Ball E. V. Shaw K. Phillips A. D. Cooper D. N. (2014). The human gene mutation database: Building a comprehensive mutation repository for clinical and molecular genetics, diagnostic testing and personalized genomic medicine. Hum. Genet. 133, 1–9. 10.1007/s00439-013-1358-4 24077912 PMC3898141

[B54] Tüttelmann F. Ruckert C. Albrecht R. (2018). Disorders of spermatogenesis. Med. Genet. 30, 12–20. 10.1007/s11825-018-0181-7 29527098 PMC5838132

[B55] Uhlen M. Oksvold P. Fagerberg L. Lundberg E. Jonasson K. Forsberg M. (2010). 'Towards a knowledge-based human protein Atlas. Nat. Biotechnol. 28, 1248–1250. 10.1038/nbt1210-1248 21139605

[B56] UniProt, Consortium (2021). 'UniProt: The universal protein knowledgebase in 2021. Nucleic Acids Res. 49, D480–D489. 10.1093/nar/gkaa1100 33237286 PMC7778908

[B57] Wang P. J. McCarrey J. R. Yang F. David C. (2001). An abundance of X-linked genes expressed in spermatogonia. J. Nat. Genet. Page 27, 422–426. 10.1038/86927 11279525

[B58] Wang P. J. Page D. C. McCarrey J. R. (2005). 'Differential expression of sex-linked and autosomal germ-cell-specific genes during spermatogenesis in the mouse. Hum. Mol. Genet. 14, 2911–2918. 10.1093/hmg/ddi322 16118233 PMC1994333

[B59] Wang X. Jin H. R. Cui Y. Q. Chen J. Sha Y. W. Gao Z. L. (2018). 'Case study of a patient with cryptozoospermia associated with a recessive TEX15 nonsense mutation. Asian J. Androl. 20, 101–102. 10.4103/1008-682X.194998 28303806 PMC5753545

[B60] Wu C. Orozco C. Boyer J. Leglise M. Batalov S. Hodge C. L. (2009). 'BioGPS: An extensible and customizable portal for querying and organizing gene annotation resources. Genome Biol. 10, R130–R138. 10.1186/gb-2009-10-11-r130 19919682 PMC3091323

[B61] Wyrwoll M. J. Temel Ş. G. Nagirnaja L. Oud M. S. Lopes A. M. Godfried W. (2020). 'Bi-allelic mutations in M1AP are a frequent cause of meiotic arrest and severely impaired spermatogenesis leading to male infertility. Am. J. Hum. Genet. 107, 342–351. 10.1016/j.ajhg.2020.06.010 32673564 PMC7413853

[B62] Yang F. Eckardt S. Leu N. A. John McLaughlin K. Wang P. J. (2008). 'Mouse TEX15 is essential for DNA double-strand break repair and chromosomal synapsis during male meiosis. J. Cell Biol. 180, 673–679. 10.1083/jcb.200709057 18283110 PMC2265566

[B63] Yang F. Lan Y. Raman Pandey R. Berger S. L. Pillai R. S. Bartolomei M. S. (2020). 'TEX15 associates with MILI and silences transposable elements in male germ cells. Genes Dev. 34, 745–750. 10.1101/gad.335489.119 32381626 PMC7263141

[B64] Yang F. Silber S. Leu N. A. Oates R. D. Marszalek J. D. Skaletsky H. (2015). 'TEX11 is mutated in infertile men with azoospermia and regulates genome-wide recombination rates in mouse. EMBO Mol. Med. 7, 1198–1210. 10.15252/emmm.201404967 26136358 PMC4568952

[B65] Yatsenko A. N. Roy A. Chen R. Lang M. Murthy L. J. Yan W. (2006). 'Non-invasive genetic diagnosis of male infertility using spermatozoal RNA: KLHL10 mutations in oligozoospermic patients impair homodimerization. J. Hum. Mol. Genet. Matzuk 15, 3411–3419. 10.1093/hmg/ddl417 17047026

[B66] Yatsenko A. N. Georgiadis A. P. Ropke A. Berman A. J. Jaffe T. Olszewska M. (2015). 'X-linked TEX11 mutations, meiotic arrest, and azoospermia in infertile men. N. Engl. J. Med. 372, 2097–2107. 10.1056/NEJMoa1406192 25970010 PMC4470617

[B23] Yeo G. Burge C. B. (2004). Maximum entropy modeling of short sequence motifs with applications to RNA splicing signals. Comput. Mol. Biol. 11 (2–3), 377–394. 10.1089/1066527041410418 15285897

[B67] Zhang X. Ding M. Ding X. Li T. Chen H. (2015). 'Six polymorphisms in genes involved in DNA double-strand break repair and chromosome synapsis: Association with male infertility. Syst. Biol. Reprod. Med. 61, 187–193. 10.3109/19396368.2015.1027014 26086992

