# Supplementary figures and images for: Genomic study of TEX15 variants: prevalence and allelic heterogeneity in men with spermatogenic failure
Source: Front Genet. 2023 May 10;14:1134849. doi: 10.3389/fgene.2023.1134849 (PMC10206016; doi:10.3389/fgene.2023.1134849)

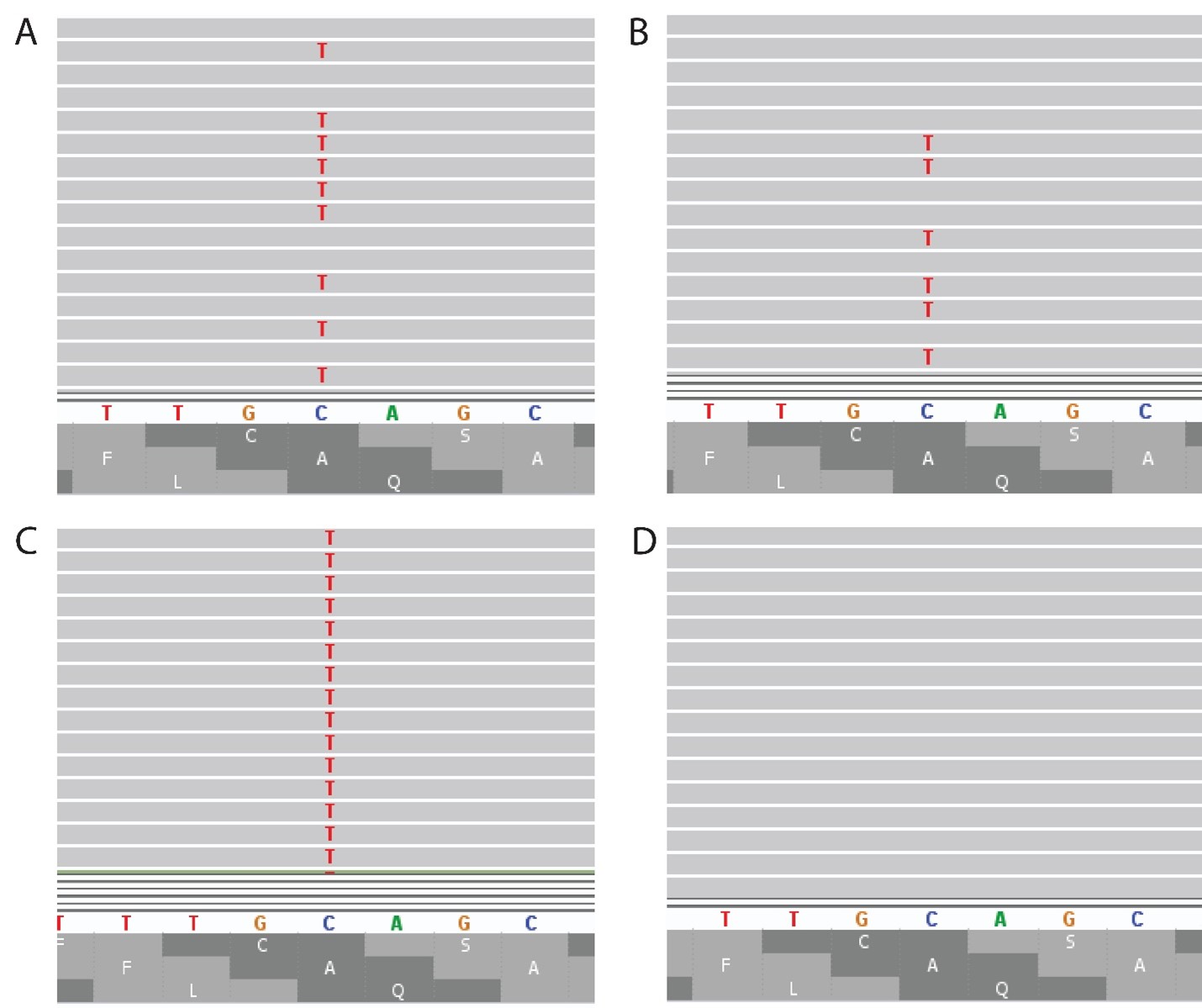

Supplement: Supplementary file 2 [file Image1.JPEG]

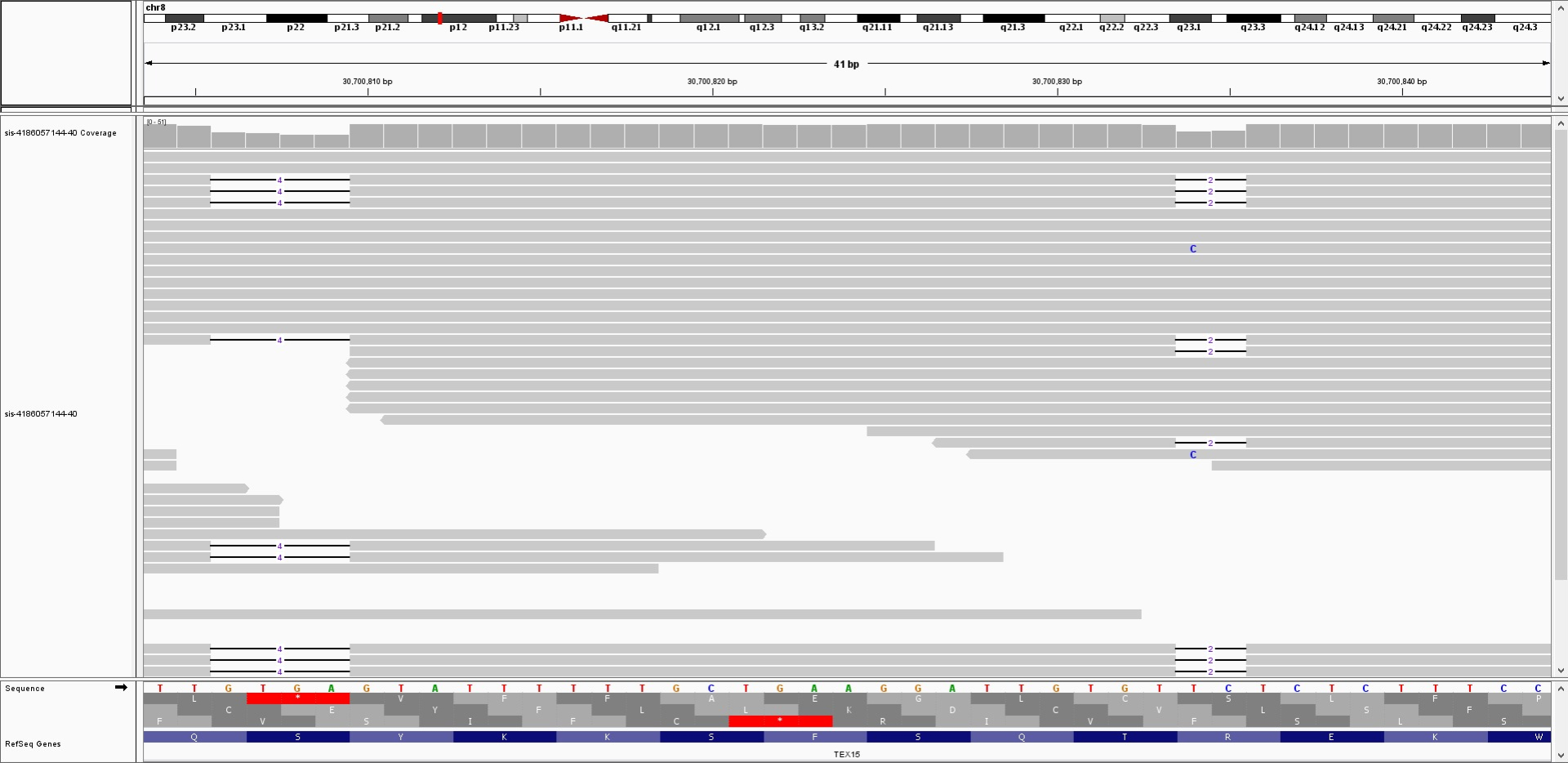

Supplement: Supplementary file 3 [file Image4.JPEG]

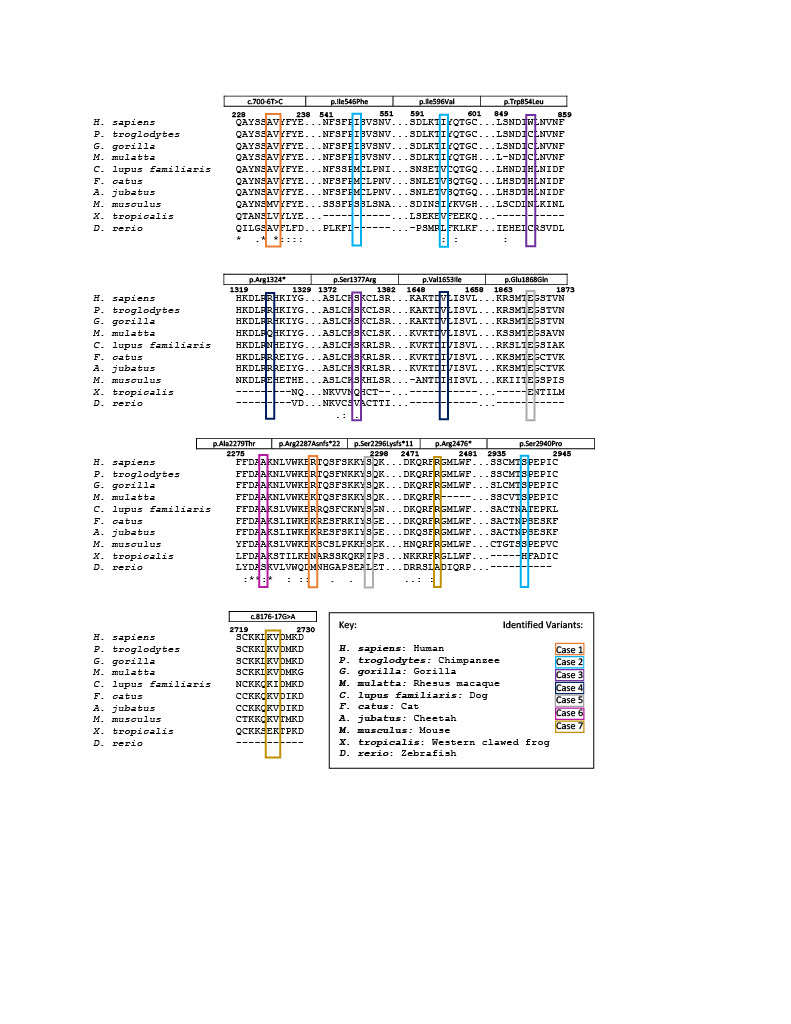

Supplement: Supplementary file 4 [file Image2.JPEG]

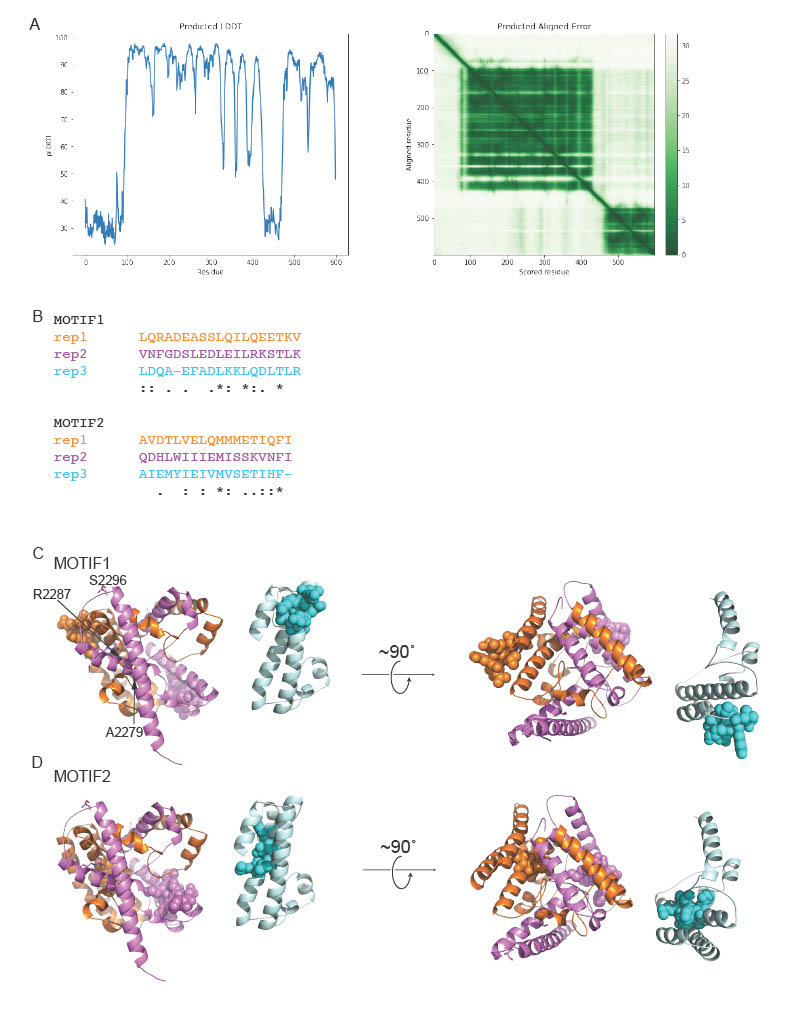

Supplement: Supplementary file 5 [file Image5.JPEG]

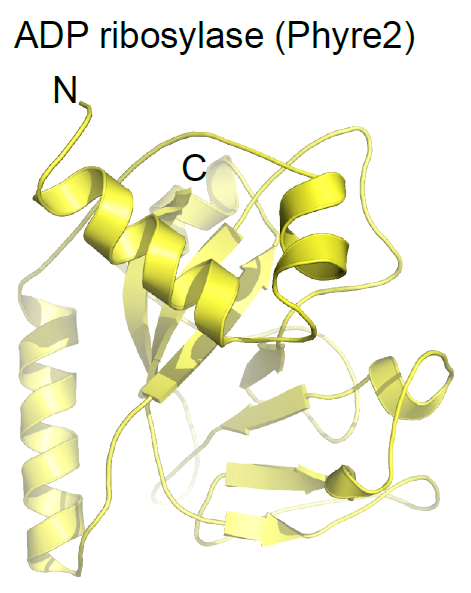

Supplement: Supplementary file 7 [file Image3.png]
